# 

**Published:** 1846-03-01

**Authors:** 


For "to Readers and Correspondents," see cover, next page.

*Erratum.*—For Hospital des Vinireris, page 225, read *Hopital des Veneriens.*

### TO READERS AND CORRESPONDENTS.

Communications have been received from "Accoucheur," Dr. Hastings, and Dr. Colvin, which are on file for publication. The review of Ricord prevented their admission into this number. Several Editorial articles were delivered to the Compositor which have been necessarily excluded, among which were notices of the very interesting Reports of the Superintendents of the Lunatic Asylums at Utica and Bloomingdale, and of Dr. Backus, of the Senate, on Lunatic Asylums and the education of Idiots.—We must ask the indulgence of those from whom favors have been received: they shall receive attention as soon as practicable. We must omit an enumeration of Publications and Exchanges received, for want of space.

---

☞ The Buffalo Medical Association will hold its regular meeting on the 3d inst., at the office of Drs. B. and G. N. Burwell.

☞ Mr. T. S. CUTTING is authorized to act as Agent for this Journal, collect subscriptions, &c.

